# StackIL10: A stacking ensemble model for the improved prediction of IL-10 inducing peptides

**DOI:** 10.1371/journal.pone.0313835

**Published:** 2024-11-14

**Authors:** Izaz Ahmmed Tuhin, Md. Rajib Mia, Md. Monirul Islam, Imran Mahmud, Henry Fabian Gongora, Carlos Uc Rios, Imran Ashraf, Md. Abdus Samad

**Affiliations:** 1 Department of Software Engineering, Daffodil International University, Daffodil Smart City (DSC), Savar, Dhaka, Bangladesh; 2 Universidad Europea del Atlántico, Santander, Spain; 3 Universidad Internacional Iberoamericana Campeche, Campeche, México; 4 Universidad de La Romana, La Romana, República Dominicana; 5 Universidad Internacional Iberoamericana Arecibo, Arecibo, Puerto Rico, United States of America; 6 Department of Information and Communication Engineering, Yeungnam University, Gyeongsangbuk-do, Gyeongsan-si, South Korea; Albert Einstein College of Medicine, UNITED STATES OF AMERICA

## Abstract

Interleukin-10, a highly effective cytokine recognized for its anti-inflammatory properties, plays a critical role in the immune system. In addition to its well-documented capacity to mitigate inflammation, IL-10 can unexpectedly demonstrate pro-inflammatory characteristics under specific circumstances. The presence of both aspects emphasizes the vital need to identify the IL-10-induced peptide. To mitigate the drawbacks of manual identification, which include its high cost, this study introduces StackIL10, an ensemble learning model based on stacking, to identify IL-10-inducing peptides in a precise and efficient manner. Ten Amino-acid-composition-based Feature Extraction approaches are considered. The StackIL10, stacking ensemble, the model with five optimized Machine Learning Algorithm (specifically LGBM, RF, SVM, Decision Tree, KNN) as the base learners and a Logistic Regression as the meta learner was constructed, and the identification rate reached 91.7%, MCC of 0.833 with 0.9078 Specificity. Experiments were conducted to examine the impact of various enhancement techniques on the correctness of IL-10 Prediction. These experiments included comparisons between single models and various combinations of stacking-based ensemble models. It was demonstrated that the model proposed in this study was more effective than singular models and produced satisfactory results, thereby improving the identification of peptides that induce IL-10.

## 1 Introduction

The immune system is made up of different types of cells and chemicals that work together to fight off infections. T cells [1] help B cells make antibodies and can also get rid of germs inside cells by turning on macrophages and killing cells that are infected with viruses. Autoimmune diseases manifest when the cells of the body are inadvertently targeted and damaged by the immune system. New research suggests that the immune system’s out-of-control function plays a part in the development of many diseases, such as cancer and autoimmune diseases [2]. Interleukin (IL)-1 receptor a (1Ra), and its other variants (IL-4, IL-10, IL-11, IL-13, IL-33, IL-35, and IL-37), as well as transforming growth factor (TGF)-*β*, can help the immune system work better when it is not working properly [3].

IL-10, a cytokine that has strong anti-inflammatory effects [4]. Mossman and Coffman were the first to clone interleukin-10 which suppresses the synthesis of cytokines by Th1 cells [5]. A number of immune cells are responsible for the production of IL-10 such as macrophages, B cells, monocytes, Th2 cells, dendritic cells and multiple T cell sub-sets [6]. IL-10 is a potent regulator of inflammation, as demonstrated by its ability to suppress TNF-*α* and IL-6 [7] production in damaged or affected tissues, while neutralization of IL-10 exacerbates pro-inflammatory cytokines [8]. IL-10 changes the expression and stimulation of receptors that recognize patterns on mast cells, which are involved in diseases associated with inflammation [9]. IL-10 is also a critical immune suppressor that modulates host-microbiota interactions, mast cell function, and the homeostatic control of infection and inflammation. IL-10, renowned for its potent anti-inflammatory attributes, can exhibit pro-inflammatory characteristics in specific contexts. The dual nature of IL-10 highlights the critical necessity of identifying IL-10-induced peptides [10]. Some studies also indicate that IL-10 immune-suppressing peptides significantly impact the development of sub-unit vaccines. However, the current experimental and computational methods pose challenges due to their prohibitive costs and the extensive time required for accurate IL-10 prediction. In response to these challenges, this work employs state-of-the-art feature extraction techniques and leverages stacking ensemble learning to enhance the prediction accuracy of IL-10. This aims to overcome the limitations associated with existing methods, contributing to the advancement of IL-10 prediction methodologies. Nagpal et al. [11] conducted an initial motif analysis and discovered many sequences that are more commonly seen in IL-10-inducing peptides as opposed to non-inducing ones. They later created several machine learning models using various feature extraction strategies, such as dipeptide composition. The Random Forest model, utilizing dipeptide composition, exhibited superior performance with a Matthews Correlation Coefficient (MCC) of 0.59 and an accuracy of 81.24%. The ILeukin10Pred study utilized an Extra Tree classifier model to detect IL-10-inducing peptides, attaining an accuracy rate of 87.5% and a Matthews Correlation Coefficient (MCC) of 0.755. Recent study suggests that combining ensemble models can improve the accuracy of peptide prediction. In order to tackle the difficulties in peptide prediction and expand upon prior investigations, this work presents a new technique known as StackIL10. This method incorporates the stacking algorithm to combine many machine learning models.

In this work, The stacking ensemble technique with amino acid composition feature encoding was used to make the StackIL10 model. There are also a number of Machine Learning models that have been taught to compare. All of the models were used to guess the IL-10-induced peptide. This work also looks at how well different methods of multiple feature encoding, like AAC, TPC, APAAC, DPC, and others, work. In terms of 10-fold-cross-validation (ACC, MCC), StackIL10 did better than other forecast methods. The testing accuracy of StackIL10 was better than that of IL-10Pred and ILeukin10Pred. Overall, the StackIL10 model that was made in this work is more accurate and works better across different situations. However, the dataset used to train and test the model was limited in size and highly imbalanced. To improve the accuracy of identifying IL-10-inducing peptides, sufficient number of positive IL-10-inducing peptide data might be required. The following is a list of the primary contributions of this study:

- Design, implementation and optimization of the StackIL10, ensemble stacking, model to predict IL-10-inducing peptide with 91.7% accuracy and 90.78% MCC.
- In the context of IL-10-induced peptide classification, this work employed nine amino acid composition-based feature extraction methods, contributing significantly to the improvement of IL-10-inducing peptide prediction.

The paper is organized into five sections in a methodical manner. Second 2 explores the literature review and provides a summary of the body of current knowledge. The Section 3 provides an in-depth exploration of the development process of StackIL10 model. The experimental results are highlighted in the next Section 4, which also offers a thorough analysis of the data and a performance comparison with other pertinent studies. Finally, Section 5 summarizes the main ideas and contributions.

## 2 Literature review

MHC peptide prediction is an important part of reasonable vaccine design because it helps to find immunogenic peptides that can make the immune system work in a safe way [12]. Different pattern recognition methods, such as motif search [13], quantitative matrix (QM), and machine learning methods, have been utilized in the past to create Interleukin-10 forecast methods. QM is a very useful method because it gives a thorough picture of how each amino acid at each position affects the binding of peptides. Most of the current T cell epitope prediction methods, on the other hand, are indirect and guess MHC class I binds [14]. These approaches are not very good at finding possible vaccine candidates and are not good for that purpose. Most of the direct and indirect ways to identify peptides such as Interleukin-10 are very hard to do and take a lot of time [15]. On the other hand, computer-based predictions can successfully cut down on the work that needs to be done in real lab experiments to find immunogenic regions [16]. Nagpal et al. [11] first did motif analysis and found a few sequences that are more common in IL-10-inducing peptides compared to non-inducing peptides. They then developed various ML models using different features extraction techniques, including dipeptide composition. The Random Forest model based on dipeptide composition worked best, with an MCC of 0.59 and 81.24% accuracy. A different study called ILeukin10Pred used an Extra Tree classifier model to find an IL-10-inducing peptide. It had an accuracy of 87.5% and a 0.755 MCC. Recent studies, including one by Singh et al. [17], have shown that stacking ensemble models can improve the performance of peptide prediction [18]. To solve the problems that come up with predicting peptides and also considering previous studies, this study created a brand-new method called StackIL10. It uses the stacking algorithm to join several machine learning models.

## 3 Materials and methods

**Fig 1:**
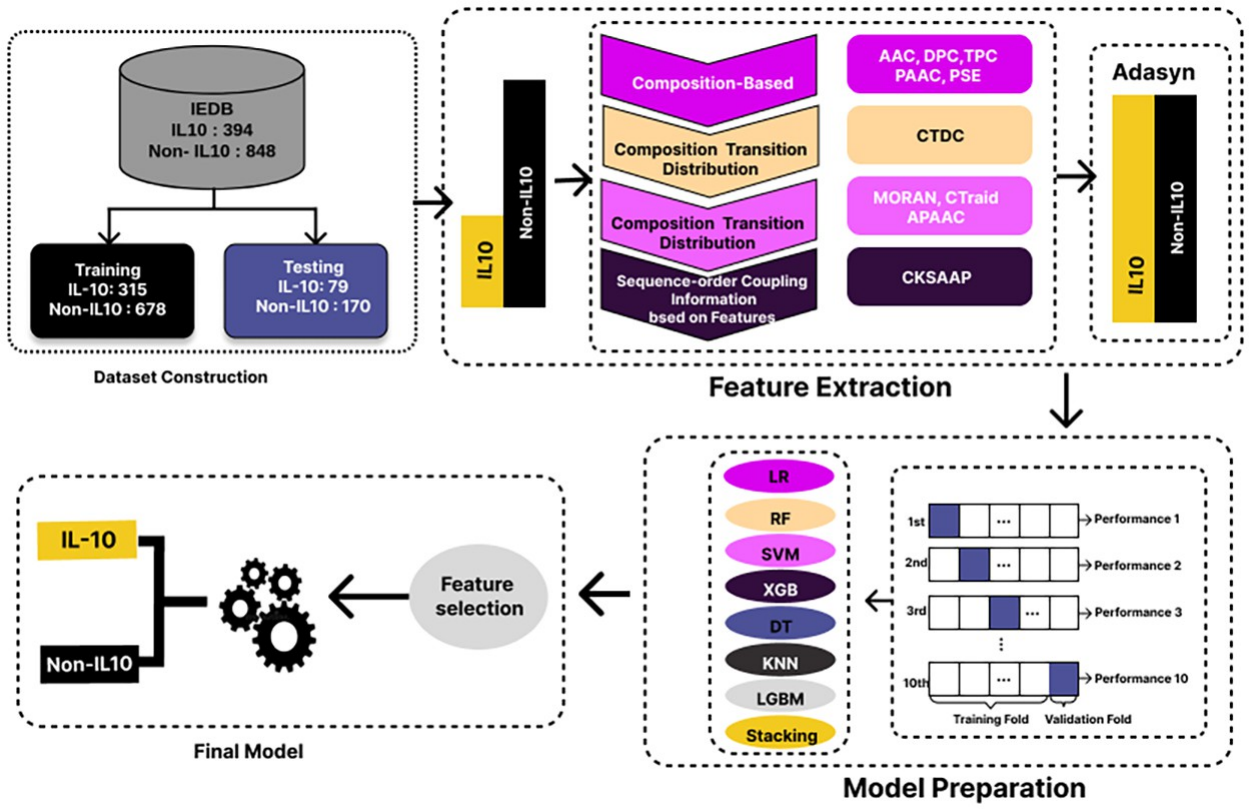
Overview of the experimental methodology for designing a IL-10-inducing peptide prediction model.

The methodology of this study involves a multi-step approach (Fig 1). First, feature extraction is initiated from a peptide sequence dataset. The dataset encompasses 394 IL-10-inducing peptides and 848 non-inducing peptides, presenting a substantial class imbalance. To address this, both ADASYN (Adaptive Synthetic Sampling) and SMOTE (Synthetic Minority Over-sampling Technique) techniques are applied for effective data balancing. Subsequently, a robust 10-fold cross-validation methodology is employed to fine-tune model hyper parameters, ensuring the generalisation capability of the predictive model. Feature selection is conducted using SHAP, an advanced technique that optimises the model by identifying and retaining the most influential features. In the final phase, the developed model demonstrates efficient prediction of IL-10.

### 3.1 Data description

To construct the prediction model for Interleukin-10 (IL-10), we utilized a benchmark dataset obtained from IEDB database by Nagpal et al [11]. The dataset contains information on antibody and T-cell epitopes obtained from experiments. In order to construct a positive dataset, IL-10-inducing peptides were created by excluding all MHC II binders that have been experimentally verified to cause the release of IL-10. Peptides that do not induce IL-10 were administered to the MHC II binders that do not have the responsibility of inducing IL-10.

The resulting dataset contained 848 non-inducing peptide sequences and 394 IL-10-inducing peptide sequences. Table 1 presents two example peptide sequences from the dataset.

**Table 1:** Overview of the dataset which represents single instance from each class.

Category	Sequence	Label
IL-10- inducing peptide	RPFERDISNVPFS	Positive
Non-IL-10-inducing peptide	SHLVEALYLVAGERG	Negative

### 3.2 Feature extraction

Feature extraction is a crucial first step in machine learning-based prediction techniques since it guarantees the effectiveness of these methods. The study used ten feature representation schemes, namely amino acid composition (AAC), dipeptide composition (DPC), composition, transition, and distribution complement (CTDC), amphiphilic pseudo-amino acid composition (APAAC), k-spaced amino acid pair composition (CKSAP), tripeptide composition (TPC), CTraid, Moran, and pseudo-amino acid composition (PAAC), PsecRAAC. The number of features for each descriptor used in the research is specified in Table 2. The ILearnPlus [19] is used to accelerate the whole computational procedure of sequence-based forecasting for DNA, RNA, and protein sequences. ILearnPlus has four functional modules built into a user-friendly interface. AAC and DPC, descriptors based on amino-acid composition, exhibited the best performance among those evaluated.

**Table 2:** Compilation of a list of descriptors, including a concise description and the number of features, utilizing iLearnPlus.

Description of Descriptor	Number of features or length of vector
Amino Acid Composition (AAC)	20
Amphiphilic Pseudo Amino Acid Composition (APAAC)	24
Composition of K-Spaced Amino Acid Pairs (CKSAAP)	1600
Conjoint Triad Descriptor of Codons (CTDC)	39
Composition, Transition and Distribution Descriptor (CTraid)	343
Dipeptide Composition (DPC)	400
Moran	2
Amphiphilic Pseudo Amino Acid Composition (PAAC)	22
Pseudo Amino Acid Composition (PsecRAAC)	4

AAC (Amino Acid Composition) descriptors quantify the frequency of occurrence of each standard amino acid within a protein sequence. For a given amino acid (*i*^*th*^), its frequency Amino is defined by the equation:

$$\begin{array}{c} \text{amino}\;\text{acid}\;\text{(i)}=\frac{\text{Amino}\;{\text{Acid}}_{i}}{\text{Length}}, \end{array}$$ (1)

In Eq 1 (Amino Acid_*i*_) represents the frequcy count of amino acid ith. By using this descriptor group, we were able to extract features based not only on AAC, but also on CKSAAP and DPC subsequently.

### 3.3 Data balancing method

The data-set contained 848 non-inducing peptide sequences and 394 IL-10-inducing peptide sequences. The dataset is highly imbalanced; an imbalanced class is a kind of classification problem in which some classes are much less common than others. After generating all the characteristics, this study proceeded to normalize the data and utilized the Adasyn synthetic sampling technique in order to avoid any bias towards the majority class, specifically peptides that do not induce IL-10. The ADASYN technique improves learning in terms of data distributions in two ways: by minimizing the bias brought on by class imbalance and by adaptively moving the classification decision boundary toward the difficult examples. The number of synthetic data points produced for each minority class data point is determined by a weight factor. The weight factor is based on the separation between the minority class data point and its nearest neighbors (Eq 2).

$$\begin{array}{c} s_{i}=x_{i}+\lambda \left(x_{k}-x_{i}\right), \end{array}$$ (2)

The difference vector between the minority class data point *x*_1_ and one of its k nearest neighbours *x*_*k*_ is a vector in n-dimensional space. The weight factor λ is a random number between 0 and 1.

### 3.4 Model designing

Diverse machine learning approaches were used to create a prediction strategy for categorising IL-10 inducing peptides. Multiple classification strategies were employed in this investigation, including Logistic Regression, Random Forest Classifier, Support Vector Classifier, Extreme Gradient, Boosting Classifier, Decision Tree Classifier, K-Nearest Neighbors, and Light Gradient Boosting Machine Classifier. A stacking classifier algorithm was also created (StackIL10) using RandomForest, XGBClassifier, DecisionTreeClassifier, support vector machine, KNeighborsClassifier, LogisticRegression, and LGBMClassifier. This algorithm performed better than other classification algorithms overall. This dataset performed satisfactorily overall. The effectiveness of the StackIL10 on additional datasets remains unclear, though. To cover all bases, it has been assessed using benchmark data from an earlier published article on IL-10Pred. The reason behind employing a stacking ensemble model in this research arises from the necessity to enhance prediction accuracy and resilience, considering the constraints of individual models. The ensemble approach effectively combines the advantageous characteristics of several classifiers, mitigating the potential problem of overfitting and augmenting the ability to generalize. Grid search and random search approaches were employed to undertake hyperparameter tuning for each base classifier. This process resulted in optimal parameter values that achieved a balance between performance and computing economy. The StackIL10 model, which combines enhanced feature extraction and a stacking ensemble model, greatly improves the accuracy of predicting IL-10 producing peptides. This demonstrates the effectiveness of the model in bioinformatics applications. In this investigation, Scikit-learn was used. Scikit-learn provides a standardized interface that focuses on tasks and allows access to a diverse set of machine learning algorithms, including supervised and unsupervised ones. Thus, the use of Scikit-learn technique facilitates the comparison of different approaches for a specific application.

#### 3.4.1 Stacking classifier

Stacking is a type of ensemble learning method used in machine learning. It uses several base models to improve the accuracy of the prediction as a whole. Another name for this is stack generalization. The methodology involves training multiple base models using the identical training dataset. Subsequently, the predictions generated by these base models are inputted into a meta-learner. This meta-learner processes the aggregated predictions to formulate the final prediction output. A stacking classifier lets us mix the best parts of different algorithms to make more accurate predictions. In StackIL10, the estimators are a combination of RF, Logistic Regression, SVM, XGBoost, LGBM, Decision Tree, and K-NN. In a stacking classifier, different models are trained on the same data, and their predictions are added together to make the end prediction, instead of just using one base model. The selection of a stacking ensemble model for IL-10 peptide prediction was motivated by the aim to leverage the complementary strengths of diverse base classifiers. Stacking allows us to integrate the distinctive capabilities of individual classifiers, enhancing predictive performance and robustness, particularly in the context of imbalanced datasets like those encountered in IL-10 peptide prediction.

#### 3.4.2 Ensemble configuration

The stacking ensemble in the StackIL10 model is structured hierarchically. Individual base classifiers, including logistic regression, decision tree, and support vector machine, XGB, LGBM, KNN, make predictions on IL-10 peptide data. These predictions are then used as input features for a meta-classifier, Logistic Regression, enhancing the overall predictive performance of the ensemble.

**Fig 2:**
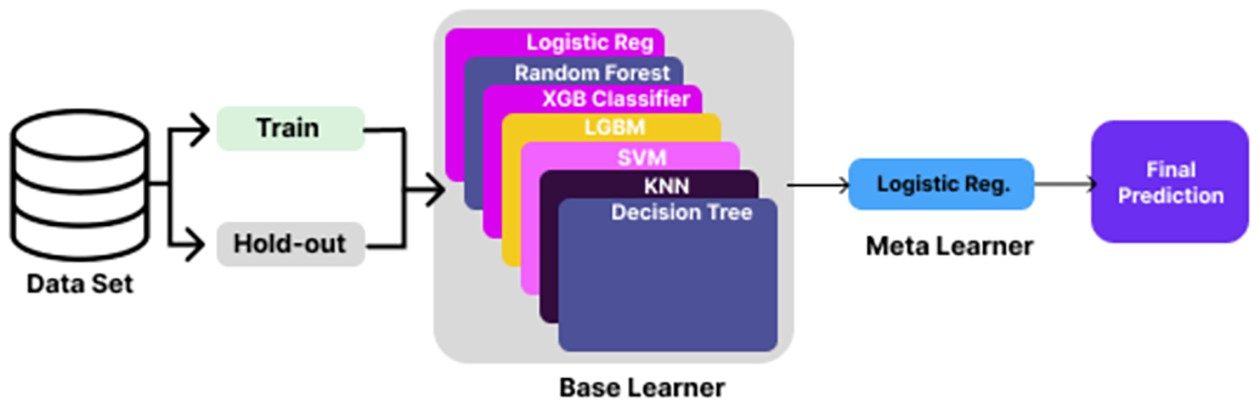
StackIL10 configuration for IL-10-inducing peptide prediction.

A dataset was used to construct an instruction set and a validation set. The stacking classifier from the ensemble module of scikit-learn was trained using the training set. In addition, the effectiveness of the model was assessed using K-fold cross-validation. Fig 2 illustrates the final StackIL10 model, which incorporates Logistic Regression as the meta-learner along with Random Forest (RF), Decision Tree (DT), Support Vector Machine (SVM), K-Nearest Neighbors (KNN), and LightGBM (LGBM) classifiers that were trained as base learners. The holdout data set was used for model validation.

#### 3.4.3 Selection of base classifier

The selection of base classifiers for the StackIL10 model was based on their diversity and complementary strengths. Multiple classifiers, including logistic regression, decision tree, and support vector machine, XGB, KNN, LGBM were chosen to capture different aspects of IL-10 peptide prediction, contributing to the overall effectiveness of the stacking ensemble. The criteria for selecting specific classifiers for the StackIL10 model included diversity in modeling approaches, individual classifier performance, and their ability to contribute varied insights to the ensemble.

#### 3.4.4 Hyperparameter tuning of base classifier

Hyperparameter tuning for both base classifiers and meta-classifier in the stacking ensemble involves grid search and randomized search techniques. For each base classifier, an individualized search space is defined, optimizing hyperparameters to enhance predictive performance. The 10-fold cross-validation is utilized to assess model generalization and prevent overfitting during hyperparameter tuning, ensuring robust performance across diverse subsets of the dataset. The RF classifier was configured with max_depth: 30, min_samples_split: 5, and _estimators: 300. And the DT classifier was configured with criterion: entropy, min_samples_split: 5, splitter: random. In addition the Support Vector Machine (**SVM**) and **LGBM** utilized default parameters.

This meticulous hyperparameter tuning process ensures that each base classifier in the StackIL10 ensemble is configured with optimal settings, contributing to the overall efficacy of the predictive model. The fine-tuned base classifiers collectively form a robust foundation for the subsequent stacking process, enhancing the model’s ability to discern IL-10-inducing peptides accurately. The implementation of the complete code of the proposed techniques is available at https://github.com/izaz-swe/StackIL10/tree/main.

## 4 Results

The evaluation of the proposed StackIL10 involves a comprehensive analysis from different perspectives. Operating as an ensemble learning-based stacking classifier, the StackIL10 model is assessed using machine learning evaluation metrics. Due to the inherent imbalance in the dataset, both the Adasyn and SMOTE oversampling methods are used. Thus, the performance is described for both situations where the data are balanced and situations where it is imbalanced. Finally, the concluding section highlights the results obtained from the combined feature analysis.

### 4.1 Performance evaluation

Performance evaluation is essential for machine learning models to evaluate their efficacy, uncover areas for improvement, and guarantee their stability for real-world implementations. Therefore, for each machine learning model, choosing the appropriate evaluation matrices is crucial. For machine learning models that make use of classification, the AUC is a standard measure of performance. A higher AUC indicates a more accurate prediction model. Here is an overview of each measure.

**Sensitivity**: The ratio of the number of positive class instances that the model correctly classifies as positive is measured by sensitivity, which is often referred to as the true positive rate (TPR). In applications such as medical diagnosis, where it is vital to prevent missing real positive cases, high sensitivity is essential. The sensitivity is defined by the Eq 3.

$$\begin{array}{c} \text{Sensitivity}=\frac{\text{TP}}{\text{TP}+\text{FN}}\times 100\% \end{array}$$ (3)

**Specificity**: Specificity, often known as the true negative rate, is a metric used to assess how well the model detects cases of the negative class. This effort aims to determine the specificity (Eq 4) with which the StackIL10 model can identify peptides that do not induce IL-10.

$$\begin{array}{c} \text{Specificity}=\frac{\text{TN}}{\text{TN}+\text{FP}}\times 100\% \end{array}$$ (4)

**Accuracy**: One commonly used metric, accuracy, indicates how well the model can predict both positive and negative occurrences. In the context of this work, accuracy is pivotal for understanding the StackIL10 model proficiency in capturing the true nature of IL-10-inducing and non-inducing peptides. For datasets without oversampling using techniques like ADASYN or SMOTE, and where classes are imbalanced, accuracy (Eq 5) serves as a holistic measure.

$$\begin{array}{c} \text{Accuracy}=\frac{\text{TP}+\text{TN}}{\text{TP}+\text{FP}+\text{TN}+\text{FN}}\times 100\% \end{array}$$ (5)

**MCC**: Taking into consideration false positives, false negatives, true positives, and true negatives, the Matthews Correlation Coefficient (MCC) offers a thorough evaluation of the StackIL10 performance that goes beyond basic accuracy. Eq 6 is useful, when there is an imbalance in the distribution of peptides that induce IL-10 and those that do not, it is quite useful.

$$\begin{array}{c} \text{MCC}=\frac{\left(\text{TP}\cdot \text{TN})-(\text{FP}\cdot \text{FN}\right)}{\sqrt{\left(\text{TP}+\text{FP})(\text{TP}+\text{FN})(\text{TN}+\text{FP})(\text{TN}+\text{FN}\right)}}\times 100 \end{array}$$ (6)

where, TP stands for true positive, TN for true negative, FP stands for false positive, and FN for false negative.

#### 4.1.1 Performance evaluation of ML models for imbalanced data

The train-test split approach was originally used to the imbalanced dataset during the training phase. Within this process, 80% of the dataset was assigned for model training, while the remaining 20% was put aside for model testing. Tables 3 and 4 provide a thorough record of the outcomes derived from the train-test division conducted on the imbalanced dataset.

**Table 3:** Evaluation of AAC feature based ML models using 10-fold CV.

Classifier	Accuracy	MCC	AUC	Sensitivity	Specificity
LR	0.6868	0.1304	0.5376	0.9458	0.1294
RF	0.7979	0.5099	0.7331	0.9104	0.5558
SVC	0.7303	0.3041	0.6109	0.9375	0.2843
XGB	0.7979	0.5183	0.7481	0.8844	0.6117
DT	0.7375	0.3884	0.6923	0.8160	0.5685
KNN	0.7367	0.3580	0.6652	0.8608	0.4695
LGBM	0.8052	0.5351	0.7554	0.8915	0.6193
**Stacking**	0.7899	0.4865	0.7170	0.9163	0.5178

**Table 4:** Evaluation of CKSAAP feature based ML models using 10-fold CV.

Classifier	Accuracy	MCC	AUC	Sensitivity	Specificity
LR	0.7327	0.3096	0.6004	0.9623	0.2386
RF	0.7979	0.5073	0.7263	0.9222	0.5305
SVC	0.7729	0.4358	0.6775	0.9387	0.4162
XGB	0.8052	0.5335	0.7527	0.8962	0.6091
DT	0.7681	0.4530	0.7208	0.8502	0.5914
KNN	0.6908	0.4912	0.7593	0.5719	0.9467
LGBM	0.8027	0.5282	0.7509	0.8927	0.6091
**Stacking**	0.8060	0.5310	0.7444	0.9127	0.5761

As indicated in Table 3, LR and KNN classifiers exhibited the least favourable performance. In particular, among the eight machine learning algorithms considered, the stacking ensemble demonstrated the highest accuracy, reaching 78.99% with 0.4865 MCC and Specificity of 0.5178.

In Table 4, during hyperparameter tuning using 10-fold cross-validation, the Random Forest and LGBM models achieved 80% accuracy on the CKSAAP feature, closely resembling the Stacking classifier. However, the Stacking Classifier outperformed, attaining the highest performance metrics: 80.60% accuracy, an AUC of 0.74, and a sensitivity of 91%.

#### 4.1.2 Performance evaluation of ML models for balanced data

The initial accuracy of the imbalanced dataset was notably low. Consequently, Adasyn and SMOTE oversampling techniques were employed to rectify the imbalance, leading to a substantial enhancement in the performance of the machine learning model. The accuracy witnessed a considerable increase, with improvements approaching nearly 10% in certain cases. As indicated in Table 5, LR and KNN, SVC classifiers exhibited the least favourable performance. Notably, among the eight machine learning algorithms considered, the stacking ensemble demonstrated the highest accuracy, reaching 88% with 0.76 MCC and Specificity of 0.85.

**Table 5:** Evaluation of AAC feature based ML models using 10-fold CV.

Classifier	Accuracy	MCC	AUC	Sensitivity	Specificity
LR	0.6319	0.2625	0.6310	0.6639	0.5980
RF	0.8702	0.7416	0.8691	0.9080	0.8302
SVC	0.7580	0.5194	0.7592	0.7182	0.8002
XGB	0.8617	0.7234	0.8612	0.8809	0.8414
DT	0.7829	0.5654	0.7827	0.7889	0.7765
KNN	0.7586	0.5507	0.7631	0.6073	0.9189
LGBM	0.8605	0.7211	0.8598	0.8844	0.8352
Stacking	0.8805	0.7616	0.8797	0.9092	0.8502

In Table 6, StackingClassifier also achieved the best performance on the APAAC feature, with 87.53% accuracy, 0.8753 AUC, and 0.75 MCC. Random Forest classifiers also performed well, achieving 86% accuracy.

**Table 6:** Evaluation of APAAC feature based ML models using 10-fold CV.

Classifier	Accuracy	MCC	AUC	Sensitivity	Specificity
LR	0.6388	0.2780	0.6389	0.6592	0.6185
RF	0.8612	0.7234	0.8612	0.8880	0.8345
SVC	0.7653	0.5328	0.7652	0.7182	0.8122
XGB	0.8347	0.6707	0.8348	0.8644	0.8052
DT	0.7218	0.4435	0.7217	0.7146	0.7289
KNN	0.8100	0.6431	0.8097	0.6757	0.9437
LGBM	0.8353	0.6726	0.8354	0.8726	0.7981
**Stacking**	0.8753	0.7510	0.8753	0.8915	0.8592

#### 4.1.3 Performance evaluation of ML models for combined feature

**Fig 3:**
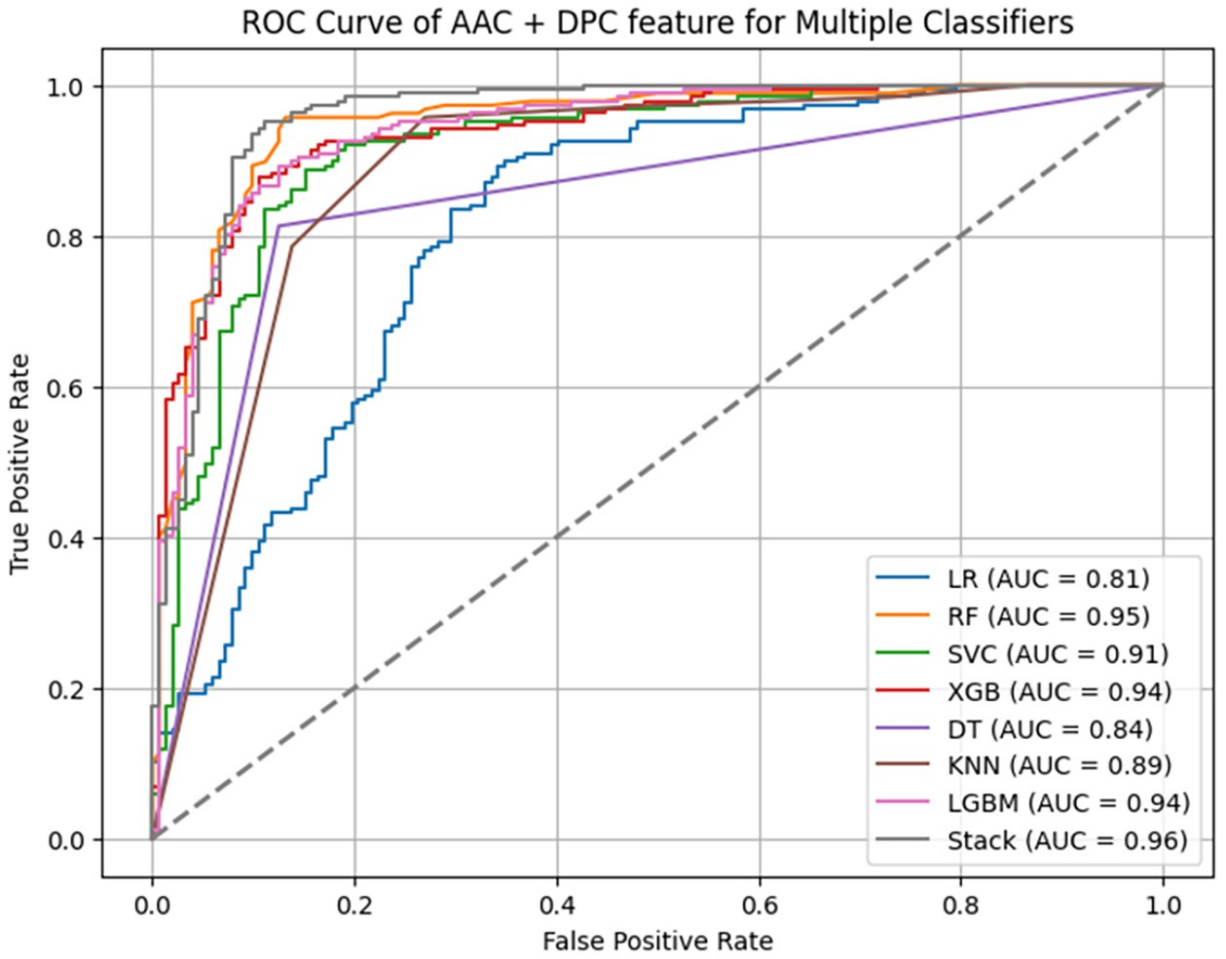
Performance comparison of different models using ROC curve.

Independent testing is a crucial step in machine learning to assess a model’s ability to generalise to unseen data and avoid overfitting. An ROC curve is a visual representation that showcases the effectiveness of a binary classification model. Fig 3 represents the independent testing performance by ROC curves of seven machine learning models using combined features: AAC, DPC. In this graph, the True Positive Rate (TPR) is plotted along the Y-axis, while the False Positive Rate (FPR) is plotted along the X-axis.

Table 7 represents, AAC + DPC feature, Logistic Regression performed the worst, with an accuracy of 63.42% and an MCC of only 0.26. However, StackingClassifier performed the best, with an accuracy of 91.74%, an MCC of 0.8330, and an AUC of 0.9165.

**Table 7:** Evaluation of AAC + DPC feature based ML models for independent testing.

Classifier	Accuracy	MCC	AUC	Sensitivity	Specificity
LR	0.634218	0.263443	0.632124	0.611842	0.652406
RF	0.876106	0.757867	0.880928	0.927632	0.834225
SVC	0.861357	0.719213	0.857093	0.815789	0.898396
XGB	0.873156	0.746891	0.875176	0.894737	0.855615
DT	0.80826	0.622108	0.812658	0.855263	0.770053
KNN	0.761062	0.55194	0.736631	0.5	0.973262
LGBM	0.876106	0.753282	0.878465	0.901316	0.855615
Stacking	0.917404	0.833028	0.916514	0.907895	0.925134

Fig 3 represents the ROC curve which helps to compare classifiers. Among the evaluated ML algorithms for AAC+DPC feature, StackingClassifier achieved the highest AUC value of 0.96, indicating superior performance in distinguishing positive and negative cases. RF also demonstrated a strong performance with an AUC of 0.95, while Logistic Regression exhibited a lower AUC of 0.81, suggesting a less effective ability to differentiate between positive and negative instances. The ROC curve analysis revealed that StackingClassifier outperformed all other ML algorithms for the AAC+DPC feature.

### 4.2 Discussion

While substantial research has been dedicated to Interleukin-10 prediction, there exists an ongoing quest for advancements in this domain. This work focuses on predicting IL-10 using an amino-acid-composition based dataset. Following dataset collection, a meticulous pre-processing stage was executed to render the data amenable to in-depth analysis. Eight supervised machine learning algorithms, specifically Decision Trees (DT), Random Forest (RF), Support Vector Machine (SVM), XGBoost, LightGBM (LGBM), and Stacking classifier (StackIL10), were employed for IL-10-inducing peptide prediction. The results of these machine learning approaches were rigorously evaluated using various performance metrics, with a particular emphasis on accuracy.

#### 4.2.1 Comparative analysis of existing work

Table 8 presents a comparative analysis of existing relevant models alongside the proposed model. The StackIL10 classifier demonstrates superior predictive performance compared to IL-10Pred and ILeukin10Pred. With the highest accuracy (0.917), StackIL10 excels in IL-10 peptide prediction, supported by its leading Matthews Correlation Coefficient (MCC) of 0.833, emphasizing a strong balance between true positives and true negatives. In particular, stackIL10 achieves the highest sensitivity (Sn) at 0.9078, demonstrating its effectiveness in identifying IL-10-inducing peptides. Although ILeukin10Pred exhibits commendable accuracy (0.875) and MCC (0.755), StackIL10 exceeds it in both metrics. IL-10Pred, while competitive, slightly lags in accuracy and MCC. In summary, the StackIL10 stacking classifier excels in accuracy, MCC, and sensitivity, highlighting the efficacy of ensemble methods, particularly stacking, in enhancing predictive outcomes in bioinformatics application.

**Table 8:** Comparison of the proposed model with existing relevant methods.

Author	Classifier	ACC	MCC	Sensitivity	Specificity
Nagpal et al.	IL-10Pred	0.812	0.590	0.797	0.819
Singh et al.	ILeukin10Pred	0.875	0.755	0.804	**0.947**
Proposed Model	StackIL10	**0.917**	**0.833**	**0.9078**	0.925

#### 4.2.2 Performance comparison among imblanced, balanced and combined feature

**Fig 4:**
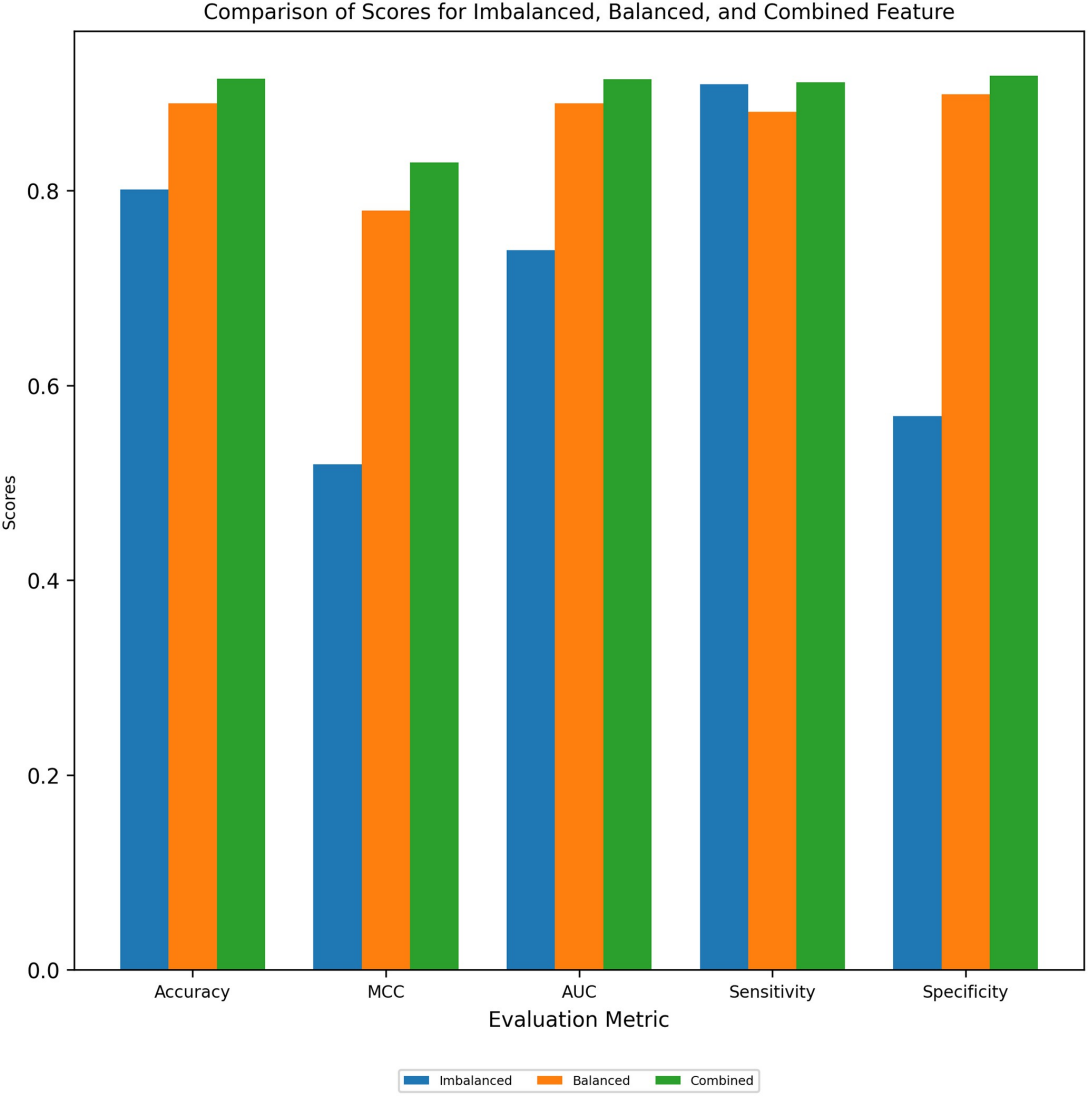
Performance comparison among imbalanced, balanced and combined feature.

Fig 4, bar chart, visually compares the Stacking model’s performance across various metrics (accuracy, MCC, AUC, sensitivity, and specificity) on imbalanced, balanced, and combined datasets. The Proposed Model achieved the highest accuracy (91.70%) and MCC (0.83) in independent testing on the Combined Feature dataset. This highlights the significant improvement gained through dataset balancing and the Stacking model’s overall effectiveness in handling diverse data scenarios.

## 5 Conclusion

Interleukin-10 (IL-10) is a cytokine with a dual role in tissue homeostasis and autoimmune diseases. IL-10 has potent anti-inflammatory properties and is essential for maintaining normal tissue homeostasis. However, defective IL-10 signaling can lead to the development of autoimmune diseases, in which the immune system mistakenly attacks the body’s own tissues. Some studies also show that guessing the immune suppressing peptide has a great effect on the production of subunit vaccines. This study introduces a new IL-10-inducing peptide prediction method, StackIL10. The model is trained on a benchmark dataset using the ILearnplus package for feature extraction and ADASYN for data balancing. A variety of machine learning models, such as RF, SVM, and LGBM, undergo training and evaluation using a 10-fold cross-validation and independent tests. The best performing models are then combined using a stacking algorithm to create the final model, StackIL10. StackIL10 is shown to achieve the best performance in the independent test set. Cutting edge tools and methods are used to create StackIL10, a new peptide prediction model for IL-10. StackIL10 was more accurate than other methods already used. However, the sample that was used to teach and test the model was not very large. To better understand and identify IL-10-inducing peptides, we need more data that have been confirmed by experiments.
